# From germline immortality to somatic rejuvenation: Unlocking the ovarian blueprint for longevity

**DOI:** 10.1371/journal.pbio.3003804

**Published:** 2026-05-26

**Authors:** Priscila Chiavellini, Vittorio Sebastiano

**Affiliations:** 1 Department‌‌ of Biological Chemistry, Center for Epigenetics and Metabolism, Stem Cell Research Center, University of California, Irvine, Irvine, California, United States of America; 2 Department of Obstetrics and Gynecology, Institute for Stem Cells Biology and Regenerative Medicine, Stanford University, Stanford, California, United States of America; Harvard University T H Chan School of Public Health, UNITED STATES OF AMERICA

## Abstract

Aging is typically framed as a one-way, irreversible accumulation of molecular damage in cells and tissues, leading to progressive functional decline. Yet mammalian reproduction, and particularly female reproduction, reveals a striking exception to this rule. Despite residing within an aging organism and within a fast-aging ovarian tissue environment, oocytes give rise to embryos that begin life with restored developmental potential and youthful molecular organization. By reframing ovarian biology as a model for rejuvenation rather than solely as a site of reproductive decline, this Essay proposes that the ovary offers a powerful blueprint for advancing the biology of aging and longevity.

## Abstract 2

Oocytes give rise to embryos that begin life with restored developmental potential and youthful molecular organisation. This Essay reframes ovarian biology as a model for rejuvenation rather than solely as a site of reproductive decline.

## Introduction

**Fig 1:**
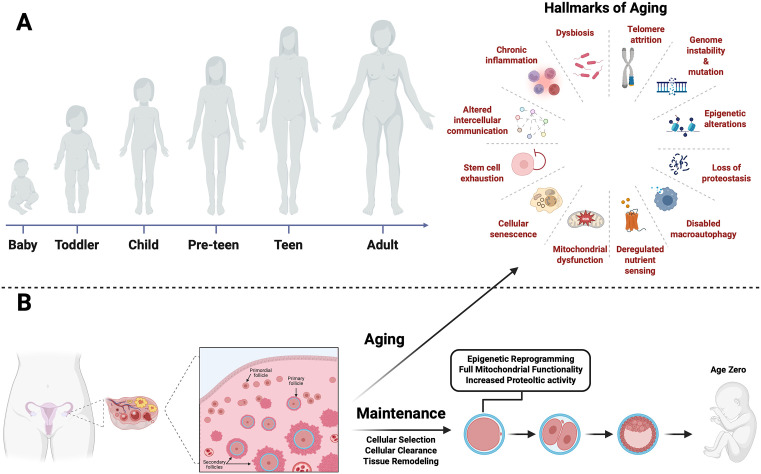
Dynamics of aging in the soma and ovary and maintenance across the life span. **A.** The body (or soma) ages over time resulting in the manifestations of the hallmarks of aging. **B.** The ovary experiences a dichotomic phenomenon. Like any other organ or tissue the ovary ages, but at the same time produces ‘immortal’ cells, the oocytes. Schematic of a female ovary in its anatomical location, illustrating the stages of follicle development. As the organism ages, the ovary exhibits the molecular hallmarks of aging. Yet it retains the capacity for self-maintenance, allowing a complete restoration of oocyte developmental potential and a reset to youthful levels, supporting the formation of a new organism. Created in BioRender. https://BioRender.com/o3o8rzr.

Aging‌‌ biology has largely focused on the gradual deterioration of somatic tissues. DNA damage accumulates, epigenetic regulation becomes unstable, mitochondria lose efficiency, senescent cells accumulate [1,2], and regenerative capacity wanes, together with many other categorized hallmarks of aging [3,4] (Fig 1). This framework is remarkably successful in explaining many features of tissues and organismal aging, yet it fails to account for one of the most fundamental processes in biology: the generation of offspring that begin life biologically young, even when derived from aged parents. Somewhere during reproduction, aging is not merely slowed but actively and effectively reversed.

The mammalian ovary embodies this paradox. It is among the first organs to exhibit functional decline, with fertility and endocrine function decreasing well before the end of life [5–8]. Nonetheless, even decades after its formation, the ovary still produces a subset of oocytes capable of generating an “age zero” offspring and supporting full embryonic and postnatal development (Fig 1). No other cell type in adult mammals, besides the oocyte, routinely performs such a comprehensive reset. The oocytes are therefore intrinsically endowed with the capacity for what we define here as rejuvenation. Throughout this Essay, we use the term rejuvenation to refer specifically to the restoration of age-associated molecular features, including epigenetic marks, mitochondrial organization, and proteostatic integrity, to a youthful state that supports full developmental competence of the offspring. This definition is distinct from clinical usage in assisted reproductive technology (ART), where rejuvenation may refer to therapeutic interventions aimed at improving reproductive outcomes in aging patients.

However, it is important to note that oocyte quality and quantity both decline significantly with age. Age-associated defects in meiotic chromosome segregation, cytoplasmic competence, and organelle function are well-documented and result in increased rates of aneuploidy and reduced embryo viability. In ART settings, even euploid embryos from older women demonstrate reduced developmental potential compared to those from younger donors. Hence, individual oocytes are not fully shielded from the aging environment, and this distinction is central to accurately framing the ovary’s rejuvenation potential.

Nonetheless, while it is undoubtably true that oocytes’ developmental competence declines with age, as we discuss below, it is remarkable to consider that whenever natural conception occurs successfully, the chronological and/or biological age of the oocytes (i.e., of the mother) is not vertically transmitted to the following generation. This is true for oocytes from younger women, middle-aged women, and, although extremely rare, also true for women that conceive naturally at more advanced ages [9].

Historically, reproductive biology and geroscience have developed as largely separate and divergent disciplines. The ovary has been studied primarily in the context of fertility and endocrine regulation, whereas aging research has focused on loss of function in somatic tissues such as the brain, muscle, immune system, and heart, including the ovary. This separation has obscured an essential insight: the ovary is not only a site of age-related decline but also the only mammalian tissue that naturally preserves an intrinsic rejuvenation capacity within its oocytes [10]. Recognizing the ovary as a biological system that both ages and rejuvenates reframes reproductive aging as a window into fundamental mechanisms of longevity rather than a narrow clinical problem.

In this Essay, we argue that the ovary, and the oocyte in particular, represent nature’s most compelling example of controlled rejuvenation. We examine how epigenetic reprogramming, mitochondrial quality control, and proteostasis operate in oocytes to preserve cellular youth. We also explore how tissue homeostasis mechanisms differ fundamentally within the ovarian niche from aging processes in somatic tissues and discuss how insights from ovarian biology can inform emerging rejuvenation strategies, including partial reprogramming, senescence modulation, and niche engineering. Finally, we discuss how the ovary itself could be a gateway to systemic rejuvenation and extended healthspan.

## The oocyte: A model of successful rejuvenation

Among mammalian cells, the oocyte is exceptional in both function and potential. Its normal biological role includes resetting biological age, allowing each generation to begin life free from the accumulated molecular damage of the previous one. This rejuvenation is not partial or superficial: it operates comprehensively across multiple layers of cellular organization and physiology, including epigenetic regulation, mitochondrial integrity, and macromolecular quality control (Fig 1).

One of the most striking features of the germline-associated rejuvenation is extensive epigenetic reprogramming. During germline development and early embryogenesis, DNA methylation patterns are globally remodeled, erasing most parental epigenetic marks and establishing new regulatory landscapes appropriate for development. Studies of human preimplantation embryos have revealed considerable changes in DNA methylation mediated by factors provided by the oocyte cytoplasm accompanying this transition, underscoring the depth of epigenetic resetting that occurs at the beginning of life [11–13]. Similarly, during fetal development, mammalian primordial germ cells (PGCs) undergo near-complete DNA demethylation, resetting parental epigenetic information while selectively retaining methylation at specific genomic regions, likely to preserve genome stability [14,15]. Subsequently, gender specific re-methylation occurs at imprinted loci [16–18].

The relative contributions of the two reprogramming waves to the removal of age-associated epigenetic marks remain unresolved. However, available evidence suggests that epigenetic restoration is most extensively achieved during the PGC wave, which exhibits the deepest genome-wide DNA methylation erasure [14] and is therefore likely the primary contributor to establishing or maintaining a youthful epigenetic ground state for the next generation.

Classic experiments of somatic cell nuclear transfer (SCNT) demonstrated that the introduction of adult somatic nuclei into enucleated oocytes can recover the developmental potential of the nuclear donor cell, indicating that the oocyte cytoplasm contains powerful reprogramming activities on any kind of “foreign” chromatin [19,20]. The ability of oocytes to reprogram even fully differentiated somatic nuclei indicates that most aging-related epigenetic alterations of the donor chromatin are fully reversible under the right conditions. After this formative collection of evidence, other landmark studies in cloned mammals showed that animals derived from aged donor nuclei can develop normally and reach adulthood. Importantly, although early cloning experiments revealed severe developmental inefficiencies due to experimental bottlenecks, subsequently refined and perfected approaches showed that cloned animals could reach expected species-specific life expectancy [21,22]. These results demonstrate that the reprogramming capacity of oocytes enables the genome of an aged somatic nucleus to support the development of organisms with lifespans consistent to species-typical norms despite the advanced age of the donor nucleus.

The finding that aged nuclei possess sufficient genomic integrity and plasticity to support the development of a new organism when exposed to the proper reprogramming environment of the oocyte laid the conceptual foundation for the development of induced pluripotent stem cell (iPSC) technology [23]. In this context, reprogramming of aged somatic cells through restoration of an embryonic-stem-cell-like epigenetic state is accompanied by a broad reversal of cellular identity and age-associated cellular phenotypes, including changes in proliferative capacity, mitochondrial function, and oxidative stress levels [24]. More recently, leveraging these compelling studies demonstrating full reversal of aging of somatic cells, we and others have shown that a tailored and tunable variation of nuclear reprogramming technology (also known as partial reprogramming, transient reprogramming, or epigenetic reprogramming of aging) can restore youthful gene regulation while preserving cellular identity [25–28].

Whether in the naturally occurring reprogramming waves during development, experimental systems such as SCNT and iPSCs, or in partial reprogramming, chromatin remodeling associated with epigenetic reprogramming is, to varying extents, accompanied by complete or partial removal of age-associated epigenetic marks. However, an important caveat must be noted here: the epigenetic resetting that occurs during germline development and early embryogenesis involves global demethylation events that erase parental epigenetic history broadly and re-establish developmental competence. This process is fundamentally tied to the generation of a totipotent zygote and is not equivalent to partial rejuvenation in somatic cells, which seeks to reverse age-associated epigenetic drift while retaining cell identity. The oocyte’s reprogramming machinery should therefore be understood as a proof of concept that age-associated epigenetic changes are reversible, not as a direct mechanistic model for how partial reprogramming operates in somatic contexts. The parallels are conceptual and motivating, but the specific molecular pathways, regulatory constraints, and biological endpoints differ substantially.

In addition to epigenetic resetting, oocytes display extraordinary mitochondrial quality control. Because mitochondria are inherited exclusively through the maternal line, the oocyte bears responsibility for transmitting a fully functional population of such organelles to the next generation [29,30]. During folliculogenesis, the number of mitochondria in oocytes increases substantially. These organelles remain relatively quiescent, relying on granulosa cells to supply energy to the oocyte, until they are activated during oocyte maturation. With a small number of mitochondrial DNA copies per mitochondrion and a distinct immature morphology, oocyte mitochondria collectively produce limited reactive oxygen species compared with somatic cells [31]. In addition, during oogenesis and early development, mitochondrial genomes undergo a genetic bottleneck, in which only a subset of mitochondrial DNA molecules is amplified, enabling rapid shifts in mitochondrial genotype frequencies across generations [32]. This process is thought to facilitate the selective propagation of high-functioning mitochondria while limiting the accumulation of deleterious mutations, a strategy starkly different from the gradual mitochondrial decline observed in aging somatic tissues.

Another distinctive feature of mitochondria in oocytes is their enrichment in long-lived proteins, as demonstrated by recent studies in mice [33,34]. These exceptionally stable proteins suggest that oocyte mitochondria are maintained through a qualitatively different proteostatic regime than somatic mitochondria, potentially contributing to organelle integrity across the extended period of oocyte dormancy. Understanding how these long-lived proteins are selectively preserved, and whether their stability contributes to or eventually limits oocyte quality with advancing maternal age, represents an important open question.

Lastly, proteostasis and macromolecular integrity further distinguish oocytes from most somatic cells [35,36]. Early embryonic development depends heavily on maternal stores of proteins and organelles, necessitating robust systems for clearing damaged components. Autophagy is strongly activated during the oocyte-to-embryo transition as a necessary step to eliminate germ-cell-specific proteins and to activate a new program of protein expression. This is essential for normal development, but it is also important as a large-scale cellular cleanup as part of rejuvenation [37]. Together, these mechanisms establish the oocyte as a model of coordinated rejuvenation, in which regulatory information, energy-producing organelles, and macromolecular quality are simultaneously restored.

It is worth acknowledging that the male germline also participates in germline renewal. Spermatogenesis continuously regenerates a population of highly specialized gametes from spermatogonial stem cells throughout adult life, and the paternal genome delivered by sperm is also subject to the major waves of epigenetic reprogramming in early embryogenesis and PGC development that collectively contribute to the generational reset of biological age [38–40]. However, the male and female germlines are fundamentally asymmetric in their contributions to rejuvenation, and this asymmetry consistently positions the oocyte as the primary driver of the process. Most critically, while sperm delivers the paternal genome, virtually the whole cytoplasmic reprogramming machinery responsible for remodeling both parental genomes after fertilization is of maternal origin. In this sense, it is the oocyte that actively executes epigenetic reprogramming not only of its own genome, but also of the paternal genome. Mitochondrial inheritance is exclusively maternal; paternal mitochondria delivered at fertilization are selectively eliminated through autophagy-mediated mechanisms [41,42], making mitochondrial quality control across generations a uniquely oocyte-dependent process. Similarly, the proteostatic stores, organellar reserves, and maternal RNA transcripts that sustain embryonic development prior to zygotic genome activation are entirely of oocyte origin. Unlike the long-lived, post-mitotic, and developmentally arrested oocyte, which must preserve molecular integrity over decades, sperm are short-lived cells that are continuously produced and rapidly replaced by spermatogonial stem cells. While this regenerative turnover mitigates some consequences of aging in the male germline, it does not confer equivalent rejuvenation depth. Indeed, paternal aging is associated with progressive accumulation of de novo mutations, DNA strand breaks, and heritable epigenetic alterations in sperm, some of which can be transmitted to offspring and influence development and health outcomes [43]. These paternal age effects parallel the well-documented consequences of maternal aging on oocyte quality and together underscore that germline rejuvenation, while operating in both sexes, is neither unlimited nor equivalent across them. Hence, while the male germline contributes to the generational reset of epigenetic information, the oocyte uniquely supplies the molecular infrastructure through which the rejuvenation of a new organism is initiated, coordinated, and executed.

## The ovarian niche: A laboratory for youth maintenance and renewal

Although the oocyte is the central promoter of rejuvenation, it does not operate in isolation. Its development and quality are inseparable from the ovarian niche, a complex and dynamic environment composed of somatic support cells, immune cells, vasculature, and extracellular signals. The ovarian niche not only nurtures oocytes but is also dynamic, characterized by continuous tissue remodeling across reproductive and post-reproductive stages.

Oogenesis consists of repeated rounds of activation, growth, and atresia. A first round of selection occurs during fetal development. Soon after the PGCs reach the gonadal ridges, they enter a phase of mitotic expansion but also massive clonal selection [44]. Following birth, most of the follicles that formed in the fetal gonads are then further eliminated before ovulation, a process often viewed as inefficient or wasteful [45]. The loss of PGCs and follicles through atresia are defining features of ovarian biology, but both the mechanistic basis and their ultimate “scope” are not yet fully understood. PGC and follicle loss likely reflects a combination of developmentally programmed attrition, resource-limited competition among cells, homeostatic tissue turnover, and, in some contexts, damage-informed elimination of compromised oocytes. These possibilities are not mutually exclusive, but they have distinct implications for whether they constitute a selective, adaptive process or primarily a passive consequence of developmental constraints. However, from a systems perspective, both processes may also have been developed during evolution to contribute to a stringently selective outcome, which could result in the enrichment of developmentally competent oocytes for reproduction. If this logic is true, it would conceptually resemble emerging strategies in aging biology that emphasize the removal of damaged or senescent cells to improve tissue function (e.g., via senolytics).

Granulosa cells have a critical role in mediating communication between the oocyte and its environment. They provide metabolic substrates, regulate meiotic progression, and respond dynamically to hormonal cues. Bidirectional signaling between oocytes and granulosa cells ensures that developmental progression is tightly coupled to cellular health. Disruption of this communication is a hallmark of ovarian aging and is associated with declining oocyte quality [46]. This idea was recently supported by a study that found that oocytes from aged mice exhibited improved developmental competence when incorporated into follicles containing young granulosa cells [47].

The ovarian niche is also notable for its remarkable plasticity. Across each reproductive cycle, the ovary undergoes extensive tissue remodeling, including angiogenesis, immune cell infiltration, and subsequent regression [48]. These processes resemble wound healing but occur repeatedly and in a regulated manner. While this cyclical remodeling supports reproductive function, it also imposes cumulative stress on the tissue, potentially contributing to the early onset of ovarian aging [49].

It is equally important to acknowledge here that the ovarian niche is not simply a rejuvenating environment. Beginning early in life, the ovary accumulates fibro-inflammatory changes driven by repeated cycles of follicular atresia, wound healing, and clearance of cellular debris from *corpora lutea*. This progressive fibro-inflammaging negatively impacts follicle development, oocyte quality, and may contribute to ovarian cancer pathogenesis [49–52]. Far from being solely a pristine rejuvenation chamber, the ovarian stroma thus represents both an asset and a problem: its dynamic remodeling capacity supports oocyte selection and renewal but also imposes cumulative structural damage that is fundamentally at odds with an idealized rejuvenation environment. These processes also represent active therapeutic targets in the field of reproductive aging. Emerging interventions such as anti-fibrotic drugs, underscore that maintaining ovarian healthspan requires active therapeutic strategies, not just reliance on intrinsic biology [53,54]. Thus, the ovary illustrates both the benefits and costs of sustained regenerative and clearance programs.

## Mechanisms of damage resistance and reset

The contrast between germline rejuvenation and somatic aging raises a fundamental question: why can the germline reset biological age while most somatic tissues cannot? Part of the answer lies in evolutionary priorities. Germ cells are under intense selective pressure to preserve genomic and functional integrity across generations, whereas somatic cells are optimized primarily for short-term organismal survival. Oocytes employ multiple layers of protection against damage accumulation, including enhanced DNA repair, suppression, and/or tight control of transposable elements expression [55], and selective elimination of defective cells. Importantly, rejuvenation in the germline is not indiscriminate. Studies of human germline epigenetic reprogramming reveal that certain genomic regions resist complete demethylation, suggesting that reset mechanisms are carefully constrained to balance rejuvenation with genomic stability [15].

Counterintuitively, the rejuvenating potential of the germline as a lineage does not render individual germ cells fully resistant to the aging environment during their development, which may impair their reproductive function, affecting primarily genome stability (i.e., aneuploidy) [56]. In aging oocytes, compromised developmental potential arises from the accumulation of age-associated molecular changes at both genomic and cytoplasmic levels. A study has shown that the genome transfer from oocytes of aged mice into young oocyte cytoplasm increased the proportion of embryos that reach the blastocyst stage [57]. In this context, it is of great interest to consider whether oocyte-mediated epigenetic resetting also declines with age, or whether it can be dissociated from developmental progression.

However, in somatic tissues, aging reflects not only damage accumulation but also the absence of such coordinated reset programs that characterize the germline. Long-lived cells such as neurons and cardiomyocytes rarely undergo global epigenetic remodeling or organelle turnover, in part because uncontrolled reset could jeopardize tissue identity or function. The germline demonstrates that these risks can be mitigated through tight regulation, offering a conceptual framework for designing safer rejuvenation strategies.

## The ovary as a systemic rejuvenation hub

**Fig 2:**
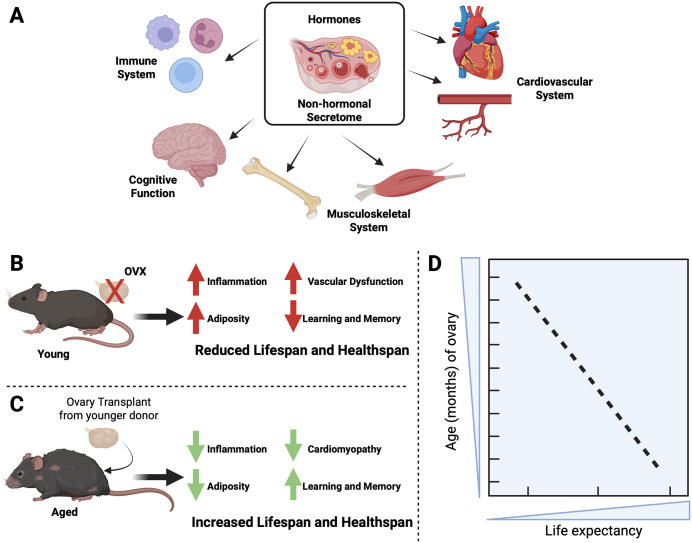
Impact of ovarian function on healthspan and life span. **A.** Schematic illustration of the organ systems influenced by the ovarian hormonal and non-hormonal secretome. **B.** Ovariectomy (OVX) in young mice reduces life span and healthspan in the animals, leading to increased inflammation, vascular dysfunction, and cognitive impairment. **C.** Transplantation of young ovaries into aged mice restores ovarian function, ameliorating systemic aging and improving life span and healthspan. **D.** An inverse correlation exists between the age of the transplanted ovary and the life expectancy of the host. Trend-line based on Fig 2 of [5]. Created in BioRender. https://BioRender.com/ryvl6fw.

Another exciting prospect offered by the ovary is the idea that its youthful physiological state or regeneration may influence the biological age of other tissues, with possible multi-organ, systemic implications (Fig 2). In rodents, ovariectomy (OVX), a standard model of abrupt ablation of ovarian signaling (surgical menopause), has a broader impact beyond fertility: it reshapes systemic aging trajectories by withdrawing cyclical ovarian endocrine output and disrupting ovary–brain–metabolism crosstalk. In female mice, long-term loss of ovarian function can shorten life span and shift tissues toward a pro-inflammatory, metabolically dysregulated state [58] (Fig 2). Complementing this, it has recently been shown that post-pubertal OVX can decouple metabolic health markers from survival, unmasking a sexually dimorphic, hormone-dependent role for hepatic mTORC2 signaling in aging [59]. These findings suggest that gonadal status could re-wire canonical longevity pathways rather than simply worsening a single disease axis. These life span effects occur alongside well-described OVX-associated phenotypes that map onto healthspan domains, from adiposity redistribution and insulin resistance to vascular dysfunction and bone loss, suggesting that ovaries act as a central, time-keeping endocrine organ, the decline of which contributes causally to multi-system aging rather than serving as a passive biomarker of it.

Strikingly, the inverse intervention, restoring youthful ovarian function late in life, can extend survival and improve age-linked pathology. An influential study showed that young ovaries transplanted into old, ovariectomized CBA mice substantially increased life expectancy at ~11 months, and that the age of the ovary predicted survival: each additional month of ovary age at the recipient’s 11-month time point reduced remaining life expectancy, implying a dose–response relationship between ovarian youthfulness and organismal longevity [5] (Fig 2). Importantly, healthspan benefits accompany the survival signal: in a histopathology-focused follow-up, transplantation of young ovaries into post-reproductive females reduced the burden of age-associated cardiomyopathy and thrombosis at death, consistent with restoration of an endogenous ‘cardioprotective milieu’ that differs from exogenous hormone replacement paradigms [60,61]. Mechanistically, the field has long noted that young ovarian grafts can reinstate estrous cyclicity in aged recipients [62], but the longevity findings suggest that cyclic hormones are not the whole story: ovarian tissue likely provides a broader secretome (e.g., steroids, peptides, metabolites, immune-modulatory cues, and axis-level feedback to the hypothalamus/pituitary) that can re-coordinate systemic aging programs, including inflammatory tone and nutrient-sensing circuitry (Fig 2).

Together, OVX and young ovary transplantation experiments support a causal model in which ovarian integrity is an active determinant of female life span and healthspan in mammals, motivating efforts to dissect ovary-derived factors and signaling circuits that might be therapeutically separable from reproduction. Notably, in this context, a few studies have shown that transplantation of germ-cell-depleted young ovaries into older mice has equal or better impact on healthspan and life span compared with transplantation of germ-cell-containing ovaries, suggesting that germ cells and somatic cells may have a partially overlapping but also divergent role on systemic aging [63,64]. In addition, solutions that might lead to endogenous ovarian rejuvenation or to the generation of youthful in-vitro-engineered ovarian tissues may have a considerable impact on systemic healthspan in women.

Although promising, several important qualifications must accompany this framework before its translational implications can be fully implemented. First, the evidence base for ovarian influence on systemic aging rests predominantly on rodent models, and the degree to which these findings translate to human biology remains an open question. Mouse reproductive aging differs from human menopause in its timeline, hormonal dynamics, and metabolic context, and the life span extension observed in young ovary transplant recipients in inbred mouse strains may not have a straightforward equivalent in the more complex physiological landscape and genetically variegated framework of human aging.

Second, the mechanistic basis of the systemic effects attributed to the ovary has not yet been resolved. While cyclic hormonal output clearly contributes, the transplantation data suggest that the ovarian secretome extends beyond classical steroids to encompass peptides, metabolites, and immune-modulatory signals [63–65], the identities and downstream targets of which remain largely uncharacterized. Distinguishing endocrine from non-endocrine contributions will be essential for developing targeted interventions that do not simply recapitulate the limitations of conventional hormone replacement.

Third, and most importantly, a conceptual distinction must be drawn between ovarian dependency and ovarian rejuvenation control. The available evidence robustly supports the view that systemic physiology is dependent on sustained ovarian signaling, and that its loss accelerates aging trajectories across multiple organ systems. It does not yet establish that the ovary actively directs or coordinates a systemic rejuvenation program in the way that the oocyte drives cellular-level reset. The ovary is best understood at present as a critical endocrine and paracrine node, the integrity of which is required for the coordination of multiple aging-related processes, rather than as an organ that autonomously drives systemic youthfulness. Reframing this distinction does not diminish the significance of the ovary as a target for healthspan extension; on the contrary, it sharpens the translational question by redirecting attention toward the specific secreted factors, signaling circuits, and tissue-level mechanisms through which ovarian integrity sustains systemic resilience, and which might be selectively preserved or restored independently of reproductive function.

## Reframing aging through the ovarian lens

Reproductive biology confronts us with a fundamental paradox at the heart of aging science. Aging is progressive, cumulative, and irreversible; yet at every generation, mammals produce offspring that begin life biologically young, even when derived from aged parents. The germline does not merely tolerate aging, it resets it. This capacity for generational renewal is not theoretical. It is a naturally occurring, tightly regulated biological program that has operated across hundreds of millions of years of vertebrate evolution, and that is concentrated, for the most part, in female reproductive biology: in the oocyte and in the ovarian environment that sustains it.

Within the ovary resides the only adult machinery that naturally preserves an intrinsic capacity for comprehensive cellular rejuvenation. The oocyte achieves this through the coordinated action of at least three interdependent processes: global epigenetic reprogramming that erases age-associated drift and re-establishes developmental competence; mitochondrial quality control that filters organelle populations through stringent inheritance bottlenecks to ensure energetic fidelity across generations; and proteostatic clearance that eliminates damaged macromolecules before they can be transmitted to the next organism. These processes are not independent molecular events but components of a unified, highly regulated renewal program. Crucially, they are also constrained: rejuvenation in the germline is coupled to quality control, and it is this coupling (i.e., the integration of reset with selectivity, and of renewal with restraint) that makes it safe. This architecture carries a clear conceptual lesson for geroscience: biological age reset in somatic tissues, whether through partial reprogramming, mitochondrial enhancement, senolytic clearance, or other interventions, will require the same principle of coordinated, bounded intervention rather than indiscriminate molecular reversal. The germline provides proof of concept that reset is possible; it does not, by itself, provide a direct molecular blueprint for how it should be safely implemented in somatic contexts, and that distinction must guide how its lessons are translated.

It is important to acknowledge that the oocyte does not operate alone in executing this rejuvenation program. While the male germline also participates (sperm contribute the paternal genome to the reprogramming process, and both germlines are subject to the major waves of epigenetic resetting during PGC development and early embryogenesis) the oocyte is, asymmetrically, the primary driver of this renewal. In fact, the cytoplasmic reprogramming machinery that remodels both parental genomes after fertilization is of exclusively maternal origin. Mitochondrial inheritance is maternal, as paternal mitochondria are selectively eliminated. The proteostatic and developmental stores that sustain embryonic life prior to zygotic genome activation are provided entirely by the oocyte. Both germlines are, moreover, subject to the consequences of aging: oocyte quality and quantity decline with maternal age, and paternal aging is associated with accumulated de novo mutations and heritable epigenetic alterations in sperm. Germline rejuvenation is therefore neither unlimited nor equivalent across the sexes. The oocyte is exceptional not because it is exempt from aging but because it retains, under appropriate conditions, the molecular infrastructure to execute a comprehensive reset despite aging, a capacity that no other somatic cell type in adults routinely possesses.

Viewed through a broader lens, ovarian aging is not only a reproductive event but a systemic one. Evidence from rodent models establishes that the ovary functions as a critical endocrine and paracrine node, the integrity of which shapes aging trajectories across multiple organ systems: loss of ovarian function accelerates inflammation, metabolic, and cardiovascular aging, while restoration of youthful ovarian signaling in aged recipients ameliorates these trajectories and extends life span. These findings motivate serious investment in ovarian healthspan as a strategy for extending female healthspan more broadly. They must, however, be interpreted with precision and caution. The available evidence robustly supports the conclusion that systemic physiology is partly dependent on sustained ovarian signaling; it does not yet establish that the ovary actively directs a systemic rejuvenation program in the way the oocyte drives cellular reset. The distinction between ovarian dependency and ovarian rejuvenation control is conceptually important: the ovary is best understood as an indispensable regulatory node, the decline of which initiates cascading dysfunction across tissues, rather than as an autonomous youth-promoting organ. Furthermore, the evidence collected thus far rests predominantly on inbred mouse models, and the translation of these findings to the more complex, genetically variegated, and physiologically complex context of human menopause remains an open and pressing question. Bridging this gap will require investment in longitudinal human cohort studies, non-human primate models, and human organoid systems capable of interrogating ovary–soma crosstalk in a physiologically relevant context. Until such data are available, the hypothesis that the ovary might functions as a systemic anti-aging regulator in women, while compelling and well-motivated, should be rigorously tested rather than be assumed as a conclusion.

The implications of this framework are, nonetheless, far-reaching. If the oocyte has evolved a machinery capable of resetting biological age under tightly controlled conditions, then biological age is, in principle, plastic. Aging therefore could be seen as a thermodynamic accumulation of molecular damage that could, under specific circumstances, be reorganized. The ovary offers, in this view, both a proof of concept and an organizational principle: rejuvenation is achievable, but it is not free. It demands quality control, selectivity, and tight regulatory architecture. These lessons, appropriately translated and rigorously validated, carry implications not only for women’s health but for the broader biology of aging in post-mitotic tissues that lack any endogenous reset capacity. Clinically, this reframing carries genuine urgency. Women spend a third or more of their lives in the post-reproductive state, and if ovarian decline contributes causally to cardiovascular, metabolic, cognitive, and skeletal aging, then extending ovarian healthspan is not a reproductive intervention but a systemic one. The development of bioengineered ovarian tissues, targeted modulators of the ovarian secretome, anti-fibrotic strategies that preserve niche integrity, and interventions that decouple the beneficial endocrine and paracrine functions of the ovary from fertility itself may collectively redefine how we approach menopause, not as an irreversible endpoint, but as a modifiable biological transition with consequences that reach far beyond reproduction.

Much of geroscience has, understandably, focused on slowing decline. The germline demonstrates that biology is capable of something more ambitious: resetting it. The ovary is the only place in the adult body where this reset still occurs; imperfectly, against a background of progressive tissue aging, constrained by fibro-inflammatory remodeling, and ultimately finite, but demonstrably real. Understanding precisely how this reset is triggered, constrained, and terminated, why it erodes with age, and which of its components can be preserved, restored, or selectively deployed in somatic systems is not peripheral to the biology of aging. If aging research is to move from mitigation toward restoration, it may need to begin not with the tissues that fail last, but with the ones that have, across all evolutionary time, always known how to begin again.

## Abbreviations

ART: assisted reproductive technology
iPSC: induced pluripotent stem cell
OVX: ovariectomy
PGCs: primordial‌‌ germ cells
SCNT: somatic cell nuclear transfer
